# Prevalence of severe adverse events among health professionals after receiving the first dose of the ChAdOx1 nCoV-19 coronavirus vaccine (Covishield) in Togo, March 2021

**DOI:** 10.1186/s13690-021-00741-x

**Published:** 2021-11-24

**Authors:** Yao Rodion Konu, Fifonsi Adjidossi Gbeasor-Komlanvi, Mouhoudine Yerima, Arnold Junior Sadio, Martin Kouame Tchankoni, Wendpouire Ida Carine Zida-Compaore, Josée Nayo-Apetsianyi, Kossivi Agbélénko Afanvi, Sibabe Agoro, Mounerou Salou, Dadja Essoya Landoh, Atany B. Nyansa, Essohanam Boko, Moustafa Mijiyawa, Didier Koumavi Ekouevi

**Affiliations:** 1 Public Health Department, University of Lomé, Lomé, Togo; 2 African Centre for Research in Epidemiology and Public Health (CARESP), Lomé, Togo; 3 Ministry of Health and Public Hygiene, Pharmacovigilance Department, Lomé, Togo; 4 Ministry of Health and Public Hygiene, Lomé, Togo; 5 Laboratory of Molecular Biology and Immunology (BIOLIM), University of Lomé, Lomé, Togo; 6 World Health Organization (WHO), Country Office of Togo, Lomé, Togo; 7 Faculty of Health Sciences, University of Lomé, Lomé, Togo; 8 Inserm Center, Institute of Public Health and Development and University of Bordeaux, 1219 Bordeaux, France

**Keywords:** Adverse event, ChAdOx1 nCoV-19, COVID-19, Vaccine, Health professionals, Togo

## Abstract

**Background:**

The coronavirus disease 2019 (COVID-19) vaccines can cause adverse events that can lead to vaccine hesitancy. This study aims at estimating the prevalence of severe adverse events (SAEs) and their associated factors among health professionals vaccinated with ChAdOx1 nCoV-19 vaccine in Togo.

**Methods:**

A cross-sectional study was conducted from March 13th to 19th, 2021 in Togo among health professionals who received the first dose of the vaccine. An online self-administered questionnaire was used to collect sociodemographic and vaccination data. SAEs were defined as one resulting in hospitalization, medical consultation, or inability to work the day following the administration of the vaccine. Data analysis were performed using R© 4.0.1 software, and a 5% significance level was considered.

**Results:**

A total of 1,639 health professionals (70.2% male) with a median age of 32 (interquartile range: 27-40) were enrolled. At least one adverse event was reported among 71.6% of participants (95% CI = [69.3-73.8]). The most commonly reported adverse events were injection site pain (91.0%), asthenia (74.3%), headache (68.7%), soreness (55.0%), and fever (47.5%). An increased libido was also reported in 3.0% of participants. Of the participants who experienced adverse events, 18.2% were unable to go to work the day after vaccination, 10.5% consulted a medical doctor, and 1.0% were hospitalized. The SAEs’ prevalence was 23.8% (95% CI = [21.8-25.9]). Being <30 years (AOR = 5.54; *p*<0.001), or 30-49 years (AOR = 3.62; *p*<0.001) and being female (AOR = 1.97; *p*<0.001) were associated with SAEs.

**Conclusions:**

High prevalence of SAEs have been observed in health professionals in Togo after ChAdOx1 nCoV-19 vaccination especially in young people and females. However, these data are reassuring as they inform on COVID-19 vaccines’ SAE management. Systematic prescription of antalgics or antipyretics could be proposed to young people who get vaccinated.

## Background

Coronavirus disease 2019 (COVID-19) was first described in China in December 2019 [1] and resulted in the declaration as a pandemic in January 2020 [2]. As of March 24, 2021, more than 120 million cases of COVID-19 infections, with more than 2 million deaths were reported worldwide [3].

No cure or vaccine was available until December 2020. Therefore, controlling the infection to prevent the spread of COVID-19 was considered the only intervention [4]. Several social and public health risk mitigation measures were proposed and implemented to reduce the spread of the virus. These measures include individual measures (frequent hand hygiene, physical distancing, and use of masks) and social distancing measures (reduction of mass gatherings, and promotion of telework) [5].

However, given that vaccination has been identified as a relevant intervention for stopping epidemics and fighting against infectious diseases, research on a COVID-19 vaccine has been promoted. At the early stage of this pandemic, the World Health Organization (WHO) and the Global Alliance for Vaccines and Immunization (Gavi) launched a pooled procurement mechanism for new COVID-19 vaccines called the COVAX Facility to ensure fair, global and equitable access to vaccines [6]. This facility also aims to end the acute phase of the COVID-19 pandemic by accelerating the development of safe and effective vaccines against COVID-19 and contributing to the development of production capacity [7]. The first vaccines received the Emergency Use Listing from WHO in December 2020 [8].

Like many African countries, Togo has joined the COVAX facility and participated in COVID-19 vaccine procurement process [6]. On March 7, 2021, Togo received the first allocation of 156,000 doses of the ChAdOx1 nCoV-19 coronavirus vaccine (Covishield) [9]. This vaccine was developed by AstraZeneca and Oxford University and produced by the Pune-based Serum Institute of India [10]. It is a viral vector vaccine that was approved for emergency use by the WHO [11]. In Togo, the vaccination campaign was launched on March 10th, 2021. According to the National Deployment and Vaccination Plan for COVID-19 vaccines, the priority target was 35,119 health professionals (HP) followed by people over 50 years of age and those living with at least one comorbidity condition (hypertension, diabetes, heart disease etc.) [12]. These groups were targeted because they are at greater risk of exposure to COVID-19.

Vaccination against COVID-19 is an essential pillar for controlling the pandemic in addition to other infection risk mitigation measures. In Togo, vaccination against COVID-19 is implemented at a time when the country is experiencing an epidemic peak with more than 600 cases per week since February 1st, 2021 compared to 100 cases per week in December 2020 [13]. As of March 28th, 2021, 9,955 COVID-19 infections cases (752 among HPs), of which, 107 deaths (2 among HPs), have been reported in Togo [13].

Despite the awareness campaign on the importance of vaccination, several factors contribute to limiting the adherence of the population to this intervention. These include: (i) the relative speed of discovery and availability of vaccines, (ii) the use of new technologies never before deployed in humans, and (iii) the lack of hindsight regarding the safety of vaccines that are available.

As a result, false information has been circulating about these new vaccines and has contributed to the growing anxiety and vaccine hesitancy associated with a fear of occurrence of long-term adverse events. In addition, the suspension of the AstraZeneca vaccine in Europe after the occurrence of thrombosis cases has amplified psychosis with popular pressure to stop the vaccination campaign in Africa including Togo.

Many studies assessed the safety of the ChAdOx1 nCoV-19 C vaccine [14, 15]. Injection site pain was the most common local side effect reported [14]. Fatigue, headache, muscle pain and joint pain are the common systemic side effects reported [14]. Thus, younger age (≤ 50 years), females, previous COVID-19 infection, and compromised health status (chronic illnesses and regular medicines uptake) seems the be associated with an increased risk of side effects [14–17].

On March 11 and 12, Togo conducted a large-scale vaccination program against COVID-19 for HP and 18,249 were vaccinated [18]. In parallel with the surveillance of adverse events following immunization which is ensured by the pharmacovigilance service of the Ministry of Health, this study was launched to quickly document adverse events to reassure the population. Our objective was to estimate the prevalence of severe adverse events (SAEs) and their associated factors among HPs in Togo.

## Methods

### Study design and setting

This study was a cross-sectional study that was conducted from March 13 to 19, 2021 in Togo.

Togo is a country of West Africa (bordered by Burkina Faso, Benin, Ghana and the Gulf of Guinea) that covers an area of 56,800 km² with an average density of 145 inhabitants per square kilometer [19]. The population was 8.08 million in 2019, of which 50.2% were women [20]. Most of the population is young (60% of Togolese are under 25 years of age), and lives in rural areas (62%) [20]. Togo’s health system has a three-level pyramid structure: central, intermediate and peripheral levels. Administrative and healthcare delivery components are associated with every level.

In March 2021, the number of HPs targeted by the vaccination campaign was estimated at 35,119 by the ministry of health. Among them, 98,2% got the first dose of ChAdOx1 nCoV-19 vaccine [21].

### Study population and sample size

The target population included all HPs who received the first dose of the ChAdOx1 nCoV-19 vaccine during the first phase of the vaccination campaign in Togo. For the purpose of this study, a health professional was defined as a professional who works or is affiliated with the health sector, including workers from health administration and logistics, clinical settings, and community health workers. The inclusion criteria for eligible participants were: (i) being a health professional aged 18 years and older; and (ii) having received the first dose of vaccine. All health professionals were invited to participate in this study. An electronic link was disseminated via an official letter from the Minister of Health and via the platforms of the health professionals’ associations. Only volunteers participated and thus where included (voluntary sampling).

The sample size of participants was calculated using a single population proportion formula with a 95% confidence level. In the absence of previous studies on the severity of the adverse events of the vaccine against COVID-19, we hypothesized that 10% of the participants would experience at least one severe adverse event with a 2% margin error and a 10% nonresponse rate. The minimum number of participants was estimated at 951.

### Data collection

A multiple-choice questionnaire was developed using items from a questionnaire of the Pharmacovigilance Department of the Togo Ministry of Health and from previous related studies found in the literature [22, 23]. The questionnaire consisted of two main sections on sociodemographic and professional characteristics (age, sex, marital status, working place) and vaccination (confidence in the vaccine efficacy, occurrence of adverse events and likelihood of getting the second dose). The self-administered Google Form® questionnaire was made available using a free online platform through the internal communication networks of the Ministry of Health. The data collection process began two days after the end of the first phase of the vaccine campaign which targeted HPs in Togo.

### Definition of the outcome variable

The main outcome was the severity of reported adverse events. Adverse events were grouped into three categories: severe (presence of adverse events resulting in self-reported hospitalization, seeking medical consultation, or inability to work the day following the administration of the vaccine), moderate (presence of adverse events without hospitalization, consultation or impact on working ability), and no side effects.

### Data management and statistical analysis

Data were imported into a Microsoft Excel database for data cleaning. Descriptive statistics were performed, and the results were presented using frequency tabulations and percentages for categorical variables. Quantitative variables were presented as medians with their interquartile range (IQR). Proportions were compared using Chi-square test or Fisher’s exact test when appropriate. Prevalence of SAEs was estimated with its 95% confidence interval (95% CI).

Univariable and multivariable logistic model regression were performed to assess factors associated with SAEs. Dependent variable was the SAE variable coded 1 when present and 0 when absent (Moderate or none adverse effect). For model building, characteristics that had a p-value <0.20 in univariable analysis were considered for the full multivariable models, which were subsequently finalized using a stepwise, backward elimination approach (p-value <0.05). Predictor variables were selected as those found to be relevant according to the literature review. Data analysis were performed using R© version 4.0.1 software, and the level of significance was set at 5%.

### Ethical considerations

Ethical approval was obtained from the ‘Comité de Bioéthique de Recherche en Santé’ (Bioethics Committee for Health Research) from the Togo Ministry of Health (No. 01/2021/CBRS). An introductory question was asked to ensure participants’ consent.

## Results

### Sociodemographic characteristics of health professionals

A total of 1,639 HPs who had received the first dose of the vaccine responded to the questionnaire. This sample represents 4.6% of the HPs vaccinated in Togo.

The median age [IQR] of the participants was 32 years [27-40] and the majority of participants were male (70.2%). Approximately half (45.8%) of the participants resided or practiced in the *Grand-Lomé* region and 54.7% were married (Table 1).

**Table 1:** Sociodemographic characteristics of health professionals vaccinated against COVID-19 in Togo, 2021 (*n* = 1,639)

	Frequency	Proportion (%)
**Age (years), Median (IQR)**	32 (27-40)	
**Age (years)**		
<30	634	38.7
30-49	862	52.6
≥50	143	8.7
**Sex**		
Female	488	29.8
Male	1151	70.2
**Health region**		
Grand-Lomé	751	45.8
Maritime	243	14.8
Plateaux	161	9.8
Centrale	104	6.4
Kara	274	16.7
Savanes	106	6.5
**Marital status**		
Single/Widowed/Divorced	742	45.3
Married	897	54.7

IQR: interquartile range

### Prevalence of adverse events

**Fig. 1:**
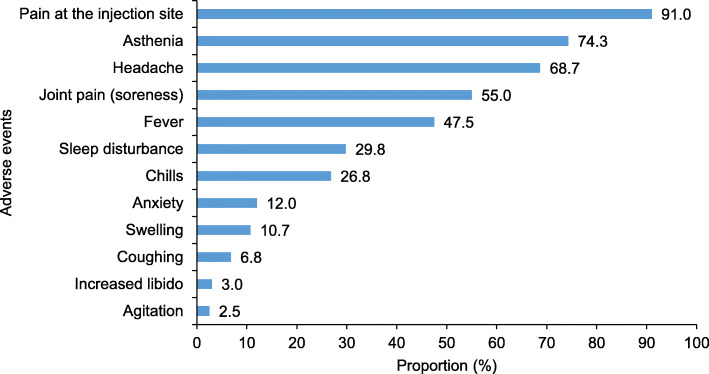
Proportion of adverse events among health professionals after vaccination against COVID-19, Togo, 2021 (*n* = 1,174)

Among the 1,639 participants, 1,174 (71.6%, 95% CI = [69.3-73.8]) reported at least one side effect. The most commonly reported adverse events were pain at the injection site (91.0%), asthenia (74.3%), headache (68.7%), body aches (55.0%), and fever (47.5%). An increase in libido was also reported in 3.0% of the participants (Fig. 1). Of the participants who experienced adverse events, 18.2% were unable to go to work the day after vaccination, 10.5% consulted a medical doctor, and 1.0% were hospitalized. Thus, the prevalence of SAEs was 23.8% (95% CI = [21.8-25.9]). This prevalence was significantly higher in female participants (33.6% versus 19.6%, *p*<0.001) and decreased with age (*p*<0.001) (Table 2).

**Table 2:** Prevalence of adverse events according to sociodemographic characteristics among health professionals, Togo, 2021

	Adverse events after COVID-19 vaccination				p-value
	**None** *N* = 442	**Moderate** *N* = 807	**Severe** *N* = 390	**Total** *N *= 1 639	
**Age (years). Median (IQR)**	34 (28-43)	32 (27-40)	30 (25-36)	32 (27-40)	<0.001*
**Age (years)**					<0.001**
<30	145 (22.9)	297 (46.8)	192 (30.3)	634	
30-49	233 (27.0)	443 (51.4)	186 (21.6)	862	
≥50	64 (44.8)	67 (46.9)	12 (8.3)	143	
**Sex**					<0.001**
Female	104 (21.3)	220 (45.1)	164 (33.6)	488	
Male	338 (29.4)	587 (51.0)	226 (19.6)	1 151	
**Health region**					0.011**
Grand-Lomé	214 (28.5)	378 (50.3)	159 (21.2)	751	
Maritime	43 (17.7)	130 (53.5)	70 (28.8)	243	
Plateaux	50 (31.)	74 (46.0)	37 (23.0)	161	
Centrale	30 (28.8)	50 (48.1)	24 (23.1)	104	
Kara	83 (30.3)	126 (46.0)	65 (23.7)	274	
Savanes	22 (20.8)	49 (46.2)	35 (33.0)	106	
**Marital status**					<0.001**
Single/Widowed/Divorced	163 (22.0)	360 (48.5)	219 (29.5)	742	
Married	279 (31.1)	447 (49.8)	171 (19.1)	897	

IQR: interquartile range; *Kruskal Wallis rank test; ** Chi-squared test

### Post vaccination treatment

Overall, medication use after vaccination was higher among participants with SAEs (*p*<0.001). The use of analgesics by participants with SAE was 62.3% vs. 39.0% for participants with moderate events and 5.2% for those reporting no events (Table 3).

**Table 3:** Use of medication after vaccination against COVID-19 according to the severity of adverse events among health professionals, Togo, 2021

	Adverse events after COVID-19 vaccination				P
	**None**	**Moderate**	**Severe**	**Overall**	
	(*n* = 442)	(*n* = 807)	(*n* = 390)	(*n* = 1,639)	
Analgesics, n (%)	23 (5.2)	315 (39.0)	243 (62.3)	581 (35.4)	<0.001*
Antipyretics, n (%)	15 (3.4)	207 (25.7)	181 (46.4)	403 (24.6)	<0.001*
NSAIDs^$^, n (%)	10 (2.3)	82 (10.2)	65 (16.7)	157 (9.6)	<0.001*
Antihistamines, n (%)	1 (0.2)	23 (2.9)	43 (11.0)	67 (4.1)	<0.001**
Vitamin C, n (%)	1 (0.2)	19 (2.4)	17 (4.4)	37 (2.3)	<0.001**
Herbal medicines/infusions, n (%)	0 (0.0)	12 (1.5)	14 (3.6)	26 (1.6)	<0.001**

^$^NSAIDs: non-steroidal anti-inflammatory drugs

*Chi-squared test; **Fisher test

### Perception after vaccination

A total of 67.5% of survey participants expressed concern about long-term adverse events, and this concern was more pronounced among women than men (74.2% vs. 64.7%, *p*<0.001). Approximately one out of ten participants (10.9%) said they were not ready to take the second dose of the vaccine (Table 4).

**Table 4:** Perceptions among health professionals after vaccination against COVID-19, Togo, 2021

	Female	Male	Overall	p
	(*n *= 488)	(*n* = 1,151)	(*n* = 1,639)	
**Afraid of long-term adverse events**				<0.001*
No	126 (25.8)	406 (35.3)	532 (32.5)	
Yes	362 (74.2)	745 (64.7)	1107 (67.5)	
**Regret to have been vaccinated**				<0.001*
No	347 (71.1)	923 (80.2)	1270 (77.5)	
Yes	141 (28.9)	228 (19.8)	369 (22.5)	
**Ready for the second dose**				0.004*
Hesitation	201 (41.2)	391 (34.0)	592 (36.1)	
No	59 (12.1)	119 (10.3)	178 (10.9)	
Yes	228 (46.7)	641 (55.7)	869 (53.0)	

*Chi-squared test

### Factors associated with the occurrence of severe adverse events

In univariable analysis, factors associated with the occurrence of SAEs included being under 30 years of age (crude Odds ratio (COR) =4.74; *p*<0.001), or 30-49 years of age (COR=3.00; *p*<0.001) and being female (COR=2.07; *p*<0.001). The same factors were associated with the occurrence of SAEs in multivariable analysis (Table 5).

**Table 5:** Factors associated with severe adverse events after COVID-19 vaccination among health professionals in Togo, 2021

	Prevalence		Binary logistic model regression					
			Univariable			Multivariable		
	n	%	COR	95%CI	p	AOR	95%CI	p
**Age (years)**								
<30	192	30.3	4.74	2.67-9.22	<0.001	5.54	2.95-11.42	<0.001
30-49	186	21.6	3.00	1.69-5.83	<0.001	3.62	1.94-7.41	<0.001
≥50	12	8.4	1.00	-	-	1.00	-	-
**Sex**								
Female	164	33.6	2.07	1.63-2.63	<0.001	1.97	1.53-2.54	<0.001
Male	226	19.6	1.00	-	-	1.00	-	-
**Having comorbidities**								
Yes	55	21.9	0.88	0.63-1.21	0.447	1.12	0.70-1.73	0.628
No	335	24.1	1.00	-	-	1.00	-	-
**History of COVID-19 infection**								
No	339	23.6	1.07	0.64-1.88	0.815	0.98	0.57-1.75	0.934
No answer	9	36.0	1.94	0.72-5.08	0.182	1.90	0.69-5.08	0.206
Yes	18	22.5	1.00	-	-	1.00	-	-

COR: Crude Odds ratio; AOR: adjusted Odds ratio; 95%CI: 95% confidence interval

## Discussion

This is the first study in Africa to report SAEs after vaccination with the ChAdOx1 nCoV-19 vaccine in the context of COVID-19 among HPs. We included 4.6% of healthcare professionals vaccinated in Togo. SAEs were reported among 23.8% of participants in the current study. Most adverse events reported were mild or severe and similar with those reported in trials of AstraZeneca [24, 25]. However, this study clearly shows that the odds of experiencing SAEs after vaccination against COVID-19 are higher in younger people (under 50 years) and women.

Based on our operational definition, approximately one-quarter of participants reported SAEs. This value could be overestimated because we included the inability to work the day after vaccination. If we consider only hospitalization and consultation of a medical doctor after vaccination, approximately 12.0% presented SAEs. In a single-blind, randomized, controlled, phase 2/3 trial (COV002), in the United Kingdom, as of October 26, 2020, 13 serious adverse events occurred during the study period, but none of those were considered to be related to the study vaccine [16]. The interim analysis of the efficacy and safety of the ChAdOx1 nCoV-19 vaccine includes data from four ongoing blinded, randomized, controlled trials performed across three countries including the UK, Brazil and South Africa, and was reported in January 2021 [24]. In this analysis, 175 SAEs occurred in 168 participants, of which three events were classified as possibly related to a vaccine: one in the ChAdOx1 nCoV-19 group, one in the control group, and one in a participant who remained masked to group allocation. A survey based on a mobile self-report questionnaire to assess the prevalence and characteristics of adverse reactions following the first dose of ChAdOx1 nCoV-19 Vaccine and BNT162b2 vaccine was conducted among healthcare workers in South Korea [26]. Of the 5,589 healthcare workers in the ChAdOx1 nCoV-19 group, the overall adverse reaction rate was 93%. About half of the ChAdOx1 nCoV-19 group reported moderate or severe grade events [26].

Several definitions exist to classify adverse events according to their severity. For example, in the COV002 trial, in the UK, SAEs were defined as substantial limitations in activity and medical intervention or the requirement of therapy [16].

We did not include, the use of medication in our definition, given that the study population included HPs who have easy access to medication and a proven tendency to self-medicate [27, 28]. Interpretations of the severity of adverse events must take into account the definition we adopted.

In our study, SAEs were more common in younger people than in people aged 50 years and older. The younger the subject was, the more severe the adverse events tended to be. This trend was described in South Korean healthcare workers were the incidence of adverse reactions was higher in those in the younger age groups [26]. The same observation has also been reported by Oxford COVID Vaccine Trial Group and seems to be related to an exaggerated immune response in young subjects [16]. In other countries, severe events following the ChAdOx1 nCoV-19 vaccine have been observed more in subjects under 55 years of age, which has prompted France among other countries to exclusively recommend this vaccine for subjects aged 55 years and older. In Togo, such recommendation is difficult to apply in our context given there is no other vaccine available to date.

Adverse events after vaccination were more pronounced in women. This observation is not unusual for vaccines in general [29] and seems to be consistent with data in the literature about COVID-19 vaccines [14, 15, 17, 26, 30, 31]. More SAEs in women have been reported in other settings and appear to be related to a stronger immune response triggered by estrogen [29] and some other unknown immunologic difference between the two sexes [32].

Most reported adverse events with the COVID-19 vaccine were mild. A sore arm was the most common, and others included headache, tiredness, and mild flu-like symptoms [33, 34]. Similar adverse events were reported in our study. However, in our study, an increase in libido was unexpectedly reported by 3.0% of HPs in both men and women. Among all UK spontaneous reports received between March 4,2021 and March 14,2021 for COVID-19 Oxford University/AstraZeneca vaccine, one case of increased libido, one of decreased libido and six cases of loss of libido were mentioned [35]. This information should be investigated to demonstrate an association with vaccination.

In regards to the perception/intention after the first dose of vaccine, two thirds of the participants expressed concern about long-term adverse events, and one out of ten declared they were not ready to take the second dose of the vaccine. These results could be explained by several concerns about ChAdOx1 nCoV-19 vaccine which arose in Europe and some African countries and resulted in the temporary suspension of the administration of this vaccine [36].

This study presents some limitations. We cannot exclude a selection bias due to our sampling method based on volunteers’ participation. This could result in an overestimated prevalence of side effects. Another limitation is that data collection was based on a declarative approach. Indeed, hospital registries where not checked to confirm HPs’ declarations. However, given that the participants were HPs, the reported effects are probably consistent with reality.

We used an operational definition to define SAE. This is a composite variable including absence from work the day after vaccination, medical consultation and hospitalization due to adverse events. This definition probably led to an overestimation of SAE, but this operational definition seems appropriate in the Togolese context.

Finally, only short-term adverse events were explored in this study. A cohort study is therefore needed for a follow-up of HPs to better document the occurrence of long-term adverse events. Nevertheless, the results from the present study are useful for designing a sensitization program in order to reassure the general population about COVID-19 vaccination. At this stage, there is no reported sign in link with thrombosis.

## Conclusions

In conclusion, despite the occurrence of SAEs, vaccination against COVID-19 remains an important strategy to fight against this pandemic. Based on these results of a high prevalence of SAEs in young people, sufficient explanation is needed to promote adherence to vaccination. Systematic prescription of antalgics or antipyretics could be proposed to young people who are willing to be vaccinated. Careful monitoring of SAEs should be performed in people under 50 years of age. If another vaccine becomes available in Togo, the ChAdOx1 nCoV-19 vaccine should be reserved for older adults.

## Data Availability

Available on request to the corresponding author.

## Abbreviations

95% CI: 95% confidence interval
CBRS: *‘Comité de Bioéthique de Recherche en Santé’* (Bioethics Committee for Health Research)
COVID-19: Coronavirus disease 2019
Gavi: Global Alliance for Vaccines and Immunization
HP: Health professional
IQR: Interquartile range
SAE: Severe adverse event
WHO: World Health Organization
